# A novel SAR reduction technique for implantable antenna using conformal absorber metasurface

**DOI:** 10.3389/fmedt.2022.924433

**Published:** 2022-08-23

**Authors:** Soumyadeep Das, Debasis Mitra, Arvind S. Chezhian, Bappaditya Mandal, Robin Augustine

**Affiliations:** ^1^Department of Electronics and Telecommunication, Indian Institute of Engineering Science and Technology, Howrah, India; ^2^Ångström Laboratory, Division of Solid-State Electronics, Department of Electrical Engineering, Uppsala University, Uppsala, Sweden

**Keywords:** absorber metasurface, biomedical application, capsule antenna module, conformal metasurface, SAR reduction

## Abstract

In this paper, a conformal absorber metasurface has been designed and used for reducing the specific absorption rate (SAR) of an implantable antenna. SAR reduction of implantable antennas is one of the significant design aspects to be considered for their use in modern-day healthcare applications. The introduction of the absorber metasurface restricts the back radiation of the antenna to control the SAR value. This technique decreases the maximum SAR value by 24% and also reduces the average SAR distribution significantly without affecting the desired antenna gain. A reduction in SAR value indicates the decrease in radiation absorption by human tissue, and thus, decreases the possibility of health hazards due to EM radiation. Later, this antenna-absorber system is designed as a capsule module for increased mobility and less-invasiveness. The redundancy of invasive surgery increases acceptance of the capsule module designs of implantable antennas and devices for various biomedical usages. *In vitro* testing of the fabricated prototype has been carried out inside a multi-layer porcine slab to verify the effectiveness of this unique SAR reduction technique.

## Introduction

Biomedical implants revolutionize the approach of the modern-day healthcare industry by continuous and accurate monitoring of patients' conditions. The implantable antennas are largely sought for as transceiver units to communicate wirelessly with and between these implants. These antennas come with the perils of electromagnetic (EM) radiation hazards over the human body (1), as the human patients are under constant exposure to EM radiation. Human tissues, being susceptible to EM radiation, are prone to numerous health hazards, such as childhood leukemia, brain tumor, albumin leakage through blood-brain barrier, etc. (2). Though no direct correlation has been achieved in the investigations carried out earlier, it is alarming that after a certain safety limit the EM radiation exposure can become problematic for human reproduction, memory as well as general tissues (3, 4).

Specific absorption rate (SAR) is the estimation of EM absorption by a unit mass of the human body. An excessive SAR value indicates greater amount of absorption by human tissue and hence more significant damage might be done to the human under consideration (5). Various studies indicate the increase in heat and temperature in the human body due to significant exposure to EM radiation. In (6), a computational framework has been discussed for studying thermoregulatory human models subjected to EM radiation under adverse environmental conditions and various types of clothing. Heat deposition is one of the known and proven effects of EM radiation and the longer duration of EM exposure, resulting in localized heating, is injurious to human tissues (7). To minimize these damages a few different approaches have already been reported. A detailed study on thermal cooling mechanisms has been carried out in (8). However, the work has been concluded with a good amount of dependency on the ambient temperature and external factors. In (9), a radiation reduction technique has been proposed to decrease the SAR value by limiting the antenna operation in half duplex mode. This obviously decreases the efficient working of the antenna.

Due to the above limitations, some direct methods of SAR reduction have been analyzed over the years. Amongst the techniques, electromagnetic shielding is widely used for wearable antennas (10). Recently, periodic structures have been used for SAR reduction of wearable antennas. An electronic band gap (EBG) structure has been operated as an in-phase reflector to reduce the SAR (11). However, the approach with a periodic structure has been largely limited to wearable antennas. Hence, the massive challenge to reduce SAR for implantable antennas remains the same.

SAR reduction of the implantable antenna is far more essential as the entire antenna is placed inside human tissue and internal human organs are in direct exposure to the antenna radiation. In a recent approach to reduce the SAR in implantable antenna, we had proposed a technique using a ferrite superstrate to reduce the discontinuities in near E-field and hence reduce the SAR (12). Though effective, this technique is comprised of a ferrite superstrate which is not biocompatible and not suitable for implanting inside the human body. A viable alternative to reduced SAR can be the introduction of a suitable periodic structure, as periodic structures are already been used for implantable antennas for gain enhancement (13).

In another aspect, placement of the implantable antenna along with other implant electronics requires excessive planning and attention. Keeping in mind the complexity and delicate nature of the human structure, an implantable antenna must be flexible, compact, and bio-compatible. Capsule-shaped antennas, being the solution to all the design challenges mentioned above, are widely used for digestive and ingestible monitoring systems (14). This less invasive alternative to the conventional implants and implantable antennas is the best suitable for the implantable scenarios, as it does not require any human surgery in the process. In (15), capsule antenna has been designed along with an RFID sensor module. Whereas, a wireless capsule endoscopy system has been designed and reported in (16). In another literature (17), a capsule antenna structure has been reported to analyze radio channel characteristics between an on-body antenna and a capsule endoscope inside the human intestine. A very recent approach to designing miniaturized antenna sensors for a capsule endoscopic module has been reported in (18). In the literature (19), a miniaturized antenna has been designed as a cylindrical capsule module for use in wireless cardiac pacemaker systems. However, to date, research communities are striving continuously toward achieving an effective technique to decrease the SAR in an implantable antenna. Keeping that need in mind, a novel SAR reduction technique, using the absorber property of metasurfaces, for implantable capsule antenna has been proposed and analyzed in this paper. SAR is the property of the human tissue mainly and not solely of an antenna, achieving reduced SAR for an implantable antenna may arises a paradoxical situation, which is discussed in section Antenna SAR reduction of this manuscript.

Absorber metasurfaces are engineered periodic structures, used for EM radiation absorption. A bianisotropic (20) metasurface has been designed and discussed in this paper to utilize the property of its magneto-electric coupling. With this property, the metasurface acts as an absorber (21) as well as prevents antenna gain reduction by stopping field scattering (22). Based on these properties we have proposed a technique, for the first time, to reduce the capsule implantable antenna SAR toward human deep tissues significantly without affecting the antenna performance. Polylactic acid (PLA), commonly known as bioplastic (23), has been employed as a substrate base for the implantable antenna and the periodic structure due to its bio-compatible and flexible nature. The periodic structure proposed in this paper acts as an absorber metasurface and is placed below the printed dipole antenna to absorb the back radiation. This unused back radiation generally increases the SAR immensely without contributing to the desired body area network (BAN) communication. Simulated results have been verified for *in vitro* conditions. Finally, we have incorporated the capsule-shaped design to make the antenna system more suitable for implantable applications as well as various ingestible practices. This capsule antenna design will be beneficial for batch production of the antenna and amalgamation of the antenna with other implantable medical devices (IMDs). The final antenna-absorber capsule module has been investigated inside a multilayer porcine slab.

Major contributions to this work can be summarized below,

- A novel topology creates a breakthrough for SAR reduction of an implantable antenna without affecting other antenna performance.
- A new bianisotropic periodic structure has been designed for this topology to utilize the absorbing property as well as reduce the field scattering.
- The antenna-absorber system has been crafted as an ingestible capsule module to enhance mobility and reduce invasiveness.

## Materials and methods

### Metasurface absorber design

**Figure 1:**
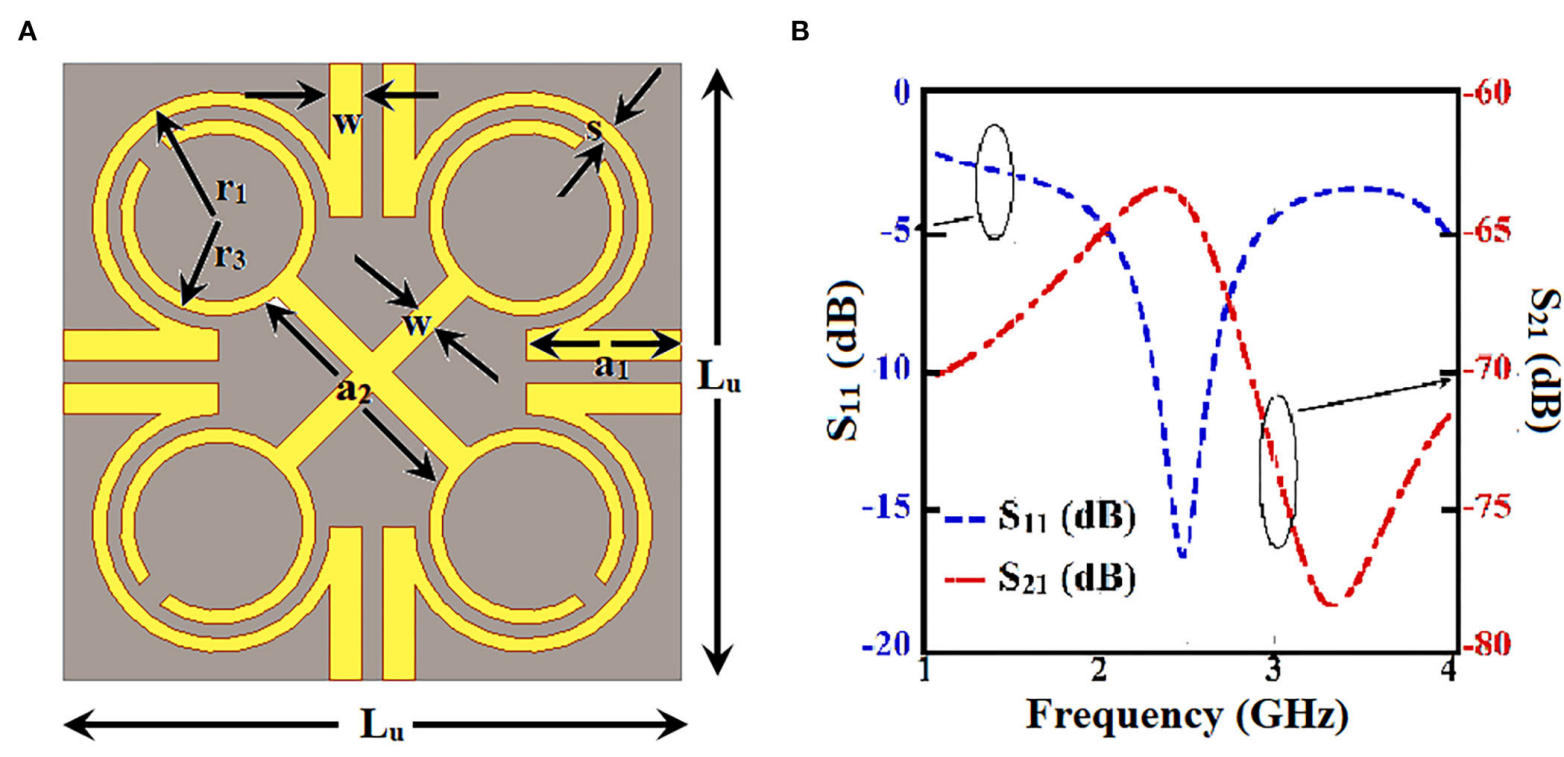
Proposed unit cell for absorber metasurface: **(A)** design top view and **(B)** *S* parameters.

**Figure 2:**
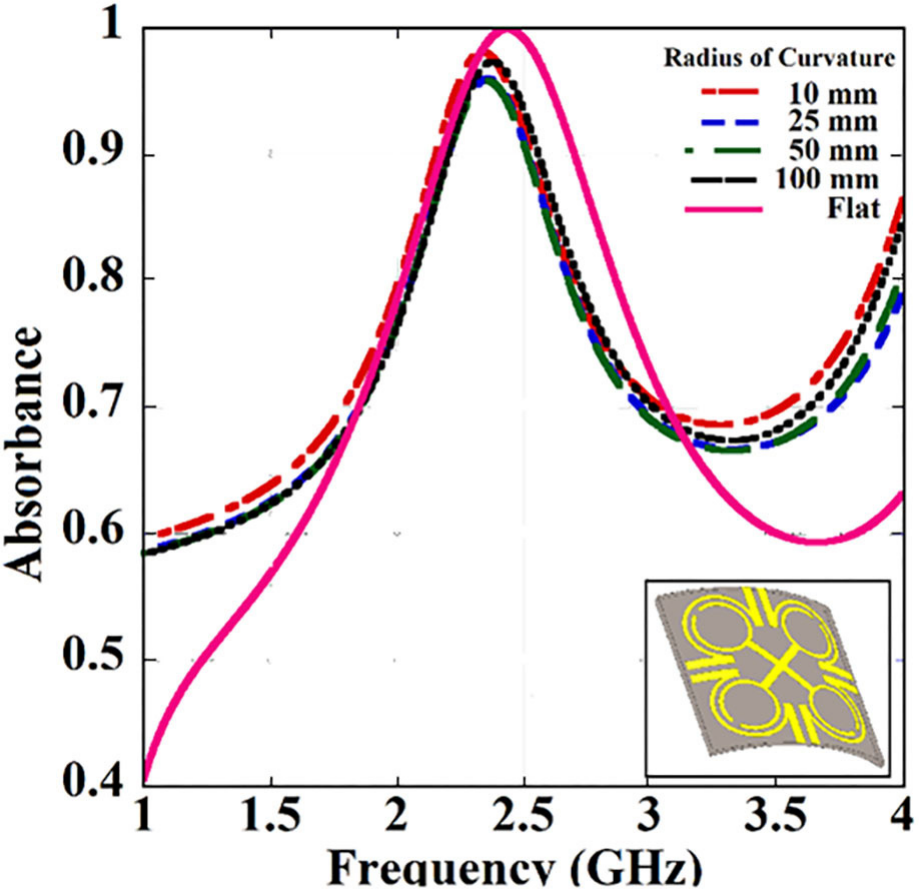
Absorbance analysis of the flexible unit cell for the different radius of curvatures (inset: Bent unit cell structure).

The metamaterial unit cell has been designed using a four Ω-shaped structures placed diagonally along with an X-shaped metallic strip (Figure 1A). In general, Ω-shaped structures show bianisotropic properties (24). The S parameters of the unit cell (Figure 1B) have been analyzed. Complex permittivity and permeability are derived from metamaterials using the effective medium approach. Complex values of permittivity and permeability typically correspond to attenuation in a medium. For designing an absorber, the attenuation must be higher. Also, the measure of absorbance can be done as, *A* = 1−*T*−*R*, where *A* is absorption, *R* is reflection, and *T* is transmission. In terms of *S* parameters, it can be deduced as, $A=1-|S_{11}{|}^{2}-{\left|S_{21}\right|}^{2}$. The proposed absorber unit cell shows high absorbance, which remains almost similar when the absorber structure is bent with the different radius of curvature (Figure 2). Validation of the conformality of the proposed absorber metasurface is thus established. The dimensions of the unit cell proposed for the absorber metasurface design are listed (Table 1).

**Table 1:** Dimensions of unit cell in mm.

**L_u_**	**a_1_**	**a_2_**	**r_1_**	**r_2_**	**w**	**S**
4.4	1.1	1.7	0.9	0.7	0.22	0.1

### Conformality analysis of the absorber

Flexibility is one of the essential requirements for IMDs and implantable antennas. The proposed absorber must have this virtue so that the entire low SAR antenna system can become conformal in design. The unit cell has been designed on PLA, which is a flexible bio-plastic. Though the designed absorber unit cell can be bent physically, the performance analysis has been done to validate whether the unit cell is flexible in nature without affecting the absorber properties. It is evident from the (Figure 2), that the absorbance of the periodic structure is mostly unaffected due to the bending with different radius of curvatures (RoCs). Hence this metasurface can be used in conformal design in implantable conditions.

### Antenna design

**Figure 3:**
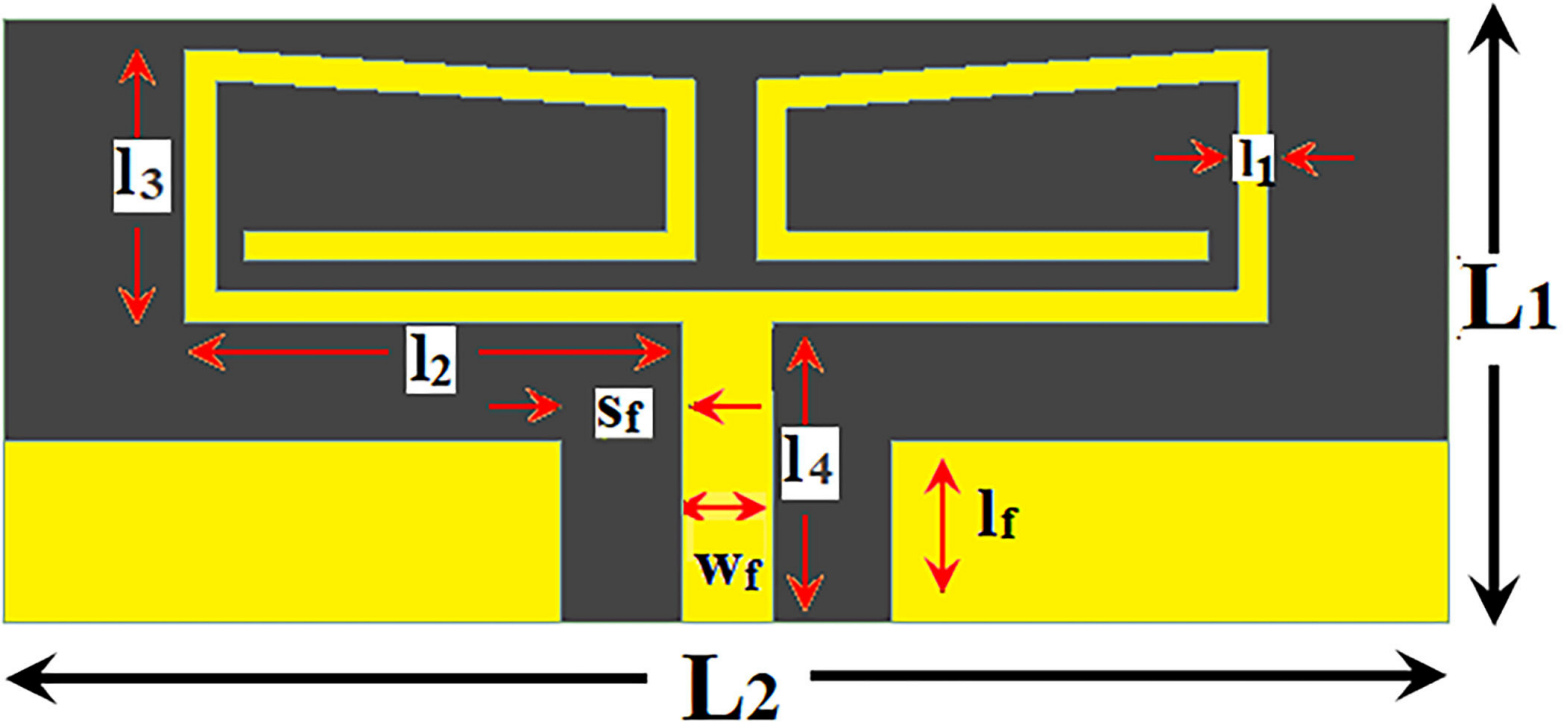
Top view of the CPW-fed printed dipole antenna in a flat surface.

A printed dipole antenna (Figure 3) is designed for implantable applications and all the relevant dimensions are presented in Table 2. This antenna with coplanar waveguide (CPW) feeding technique is etched over a PLA slab of 0.5 mm thickness. At 2.4 GHz, the PLA has dielectric constant (ε) 2.72 and loss tangent (tan δ) 0.008 (25). The final proposed antenna system is aimed to be placed inside the human torso and thus analyzed with high frequency structure simulator (HFSS 19.2) by using a single layer muscle model at first.

**Table 2:** Dimension of implantable antenna in mm.

**L_1_**	**L_2_**	**l_1_**	**l_2_**	**l_3_**	**l_4_**	**s_f_**	**w_f_**	**l_f_**
10	24	0.5	8.25	4.5	5	2	1.5	3

**Figure 4:**
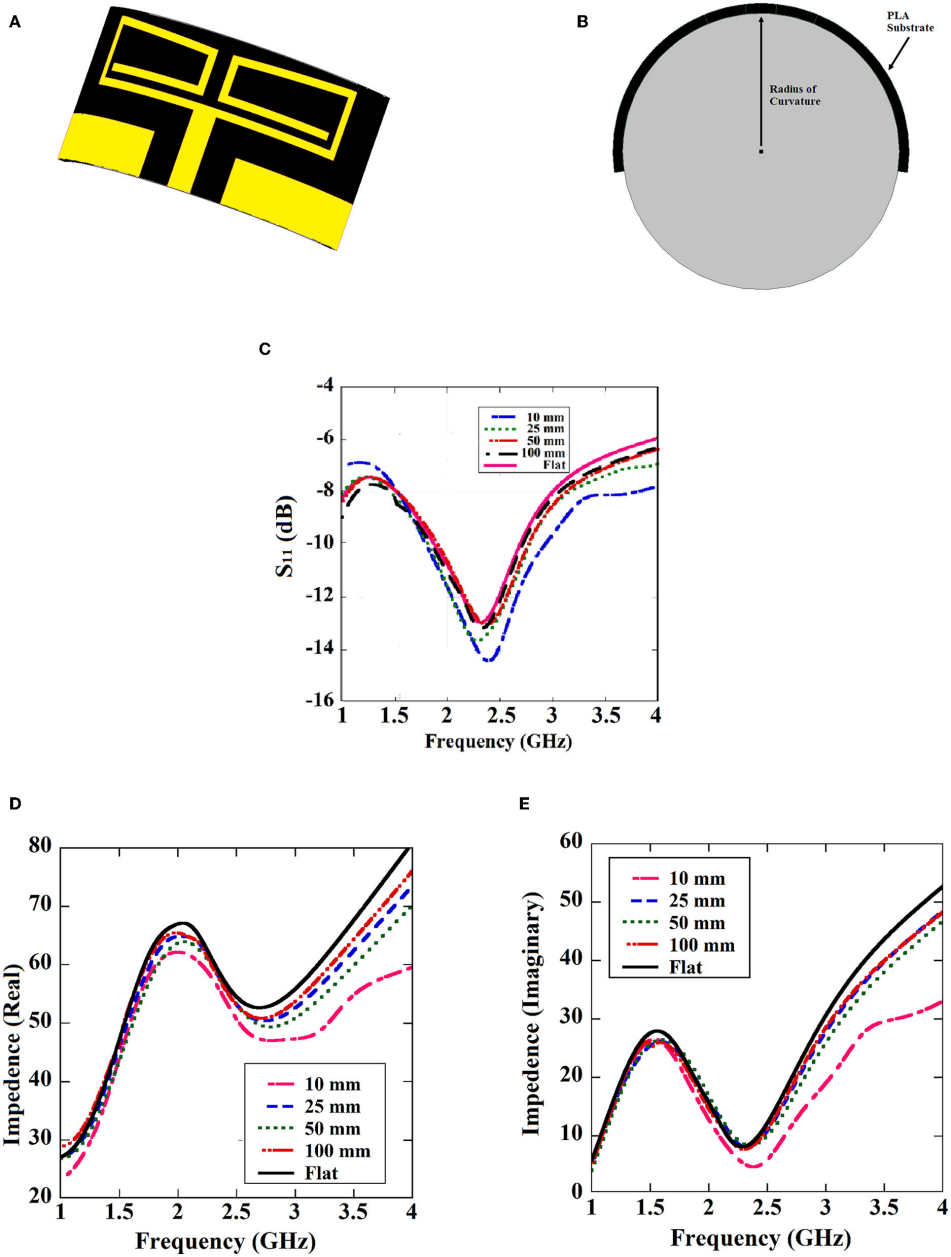
Bending analysis of the proposed antenna; **(A)** antenna structure, **(B)** radius of curvature of the bent antenna, **(C)** return loss characteristics of the curved antenna for various RoCs, **(D)** antenna impedance real, and **(E)** antenna impedance imaginary.

**Figure 5:**
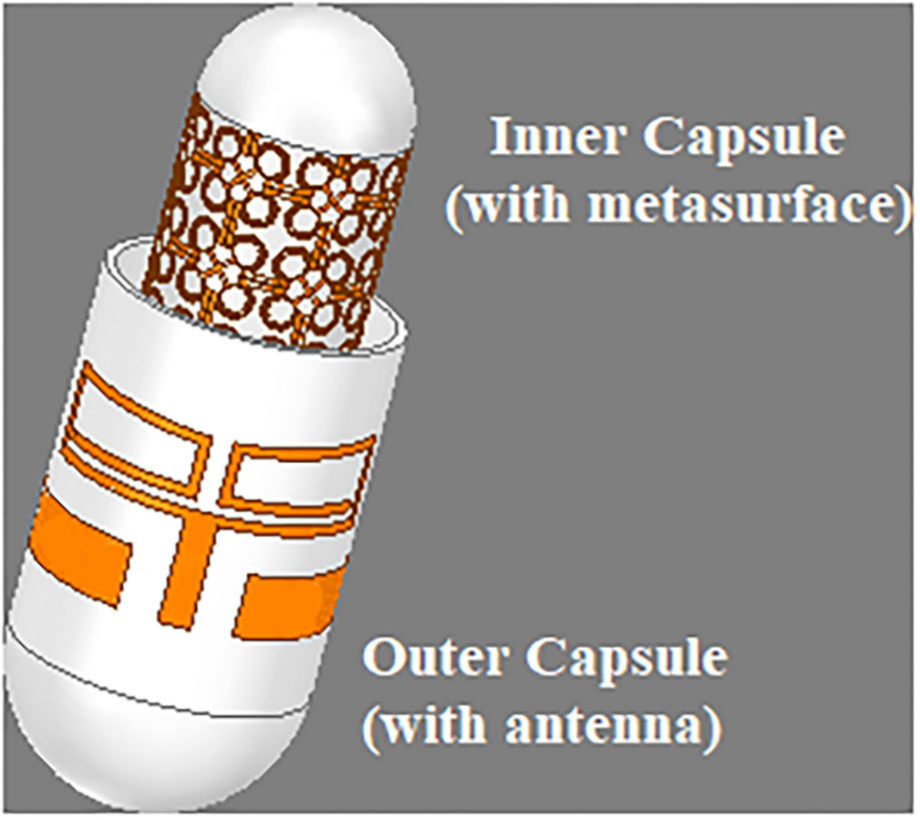
Schematic model of the proposed antenna-absorber capsule module.

**Figure 6:**
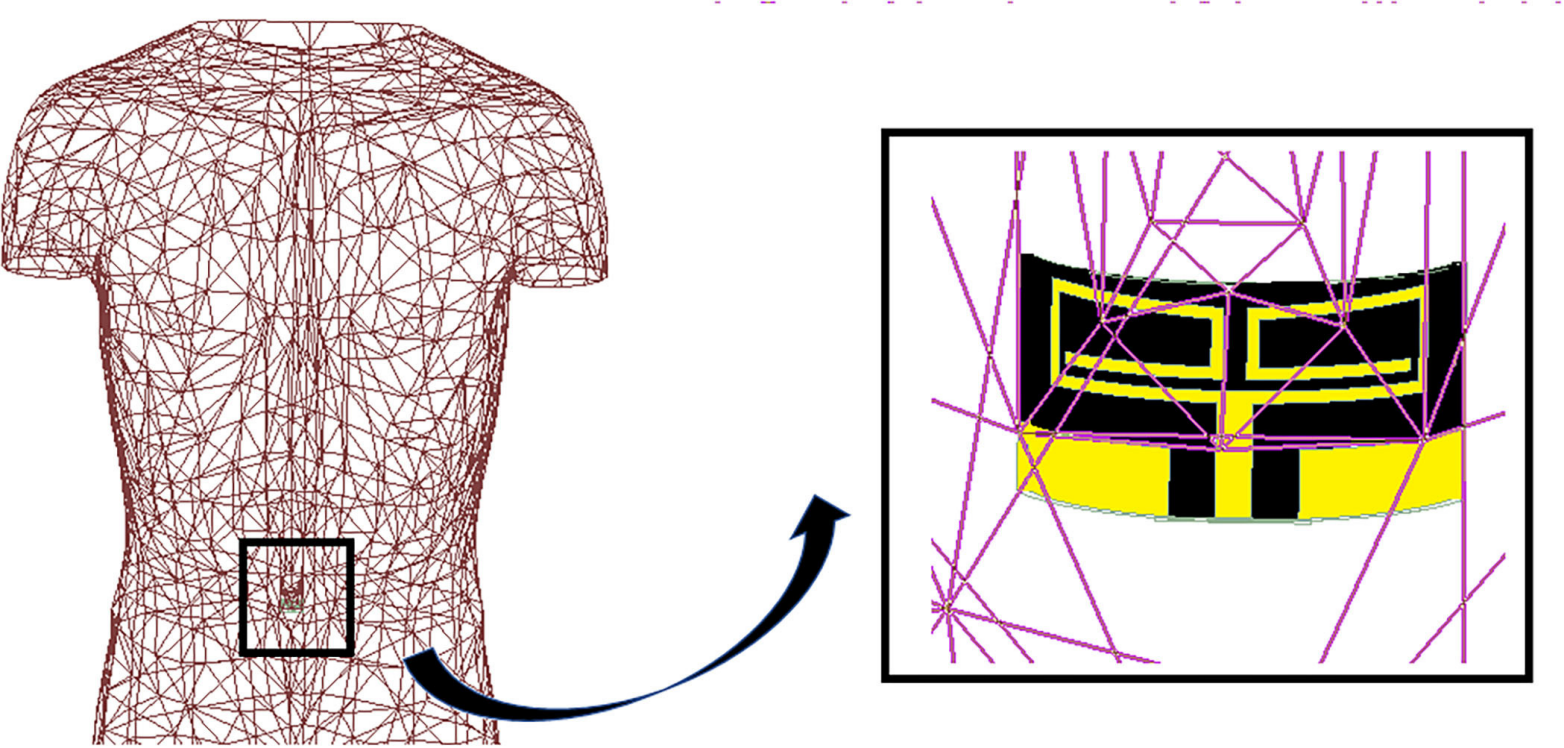
Antenna placement inside the human torso.

**Figure 7:**
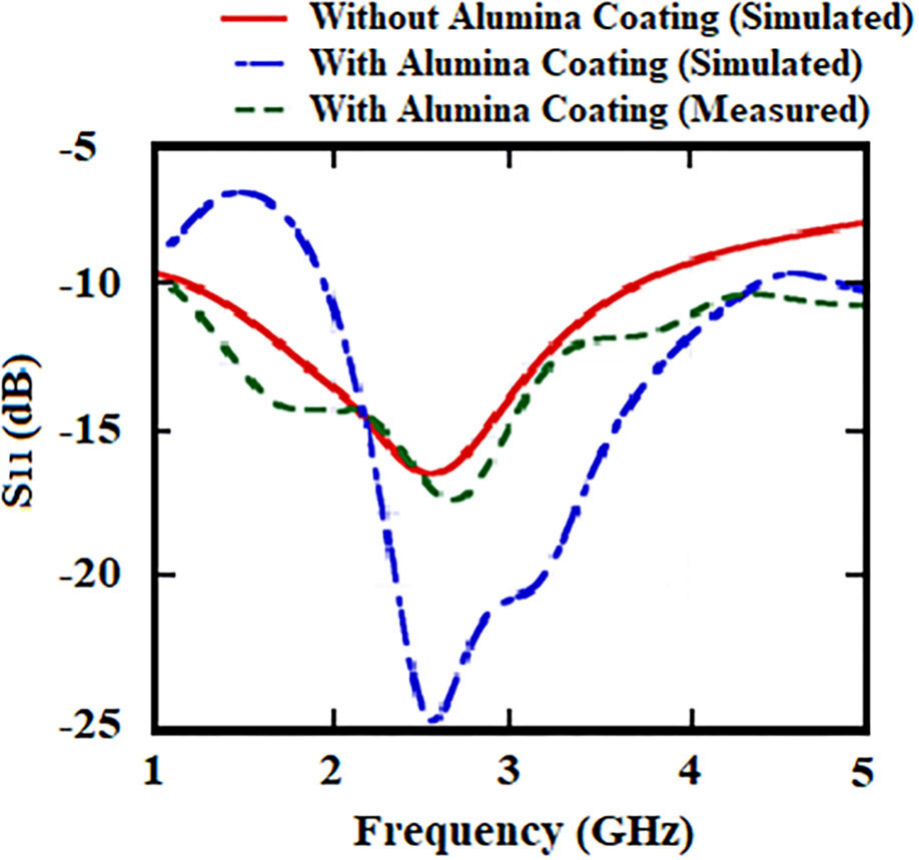
Simulated and measured return loss characteristics of the capsule antenna module.

The printed dipole antenna is analyzed for flexibility over a range of various RoCs. The resonant frequency and impedance matching have not been affected due to the structural bending of the proposed antenna (Figures 4A–E). Depending on this analysis, the RoC of the proposed antenna is optimized to 10 mm. This measure makes the antenna conformal and suitable for capsule formation. The capsule module for the antenna-absorber system has been proposed (Figure 5) to make the implanting process less-invasive. This module consists of two capsules, i.e., inner capsule and outer capsule, made of PLA. The absorber metasurface is placed over the inner capsule. Then the inner capsule is kept inside the outer capsule. The printed dipole antenna has been attached to the exterior surface of this outer capsule. As per the antenna optimization, the diameter of the outer capsule is 9–10 mm approximately and accordingly diameter of the inner capsule is 8 mm. This capsule module is intended to be consumed orally and hence it will traverse the entire Gastro Intestinal (GI) tract. Various parts of GI tract are having different diameters, such as the esophagus having an average diameter of around 3 cm, whereas small intestine and large intestine have the average diameter of 2.5 and 4.8 cm, respectively. Hence the proposed capsule antenna can go through the GI tract and radiates accordingly. The alignment of the capsule module will definitely be altered during the journey due to peristaltic motion inside the GI tract, but it will not affect the relative placement of absorber and the antenna as these two are combined as a composite capsule module. Hence always the back radiation of the antenna will be absorbed during the entire process irrespective of the alignment of the capsule module inside the GI tract. In other hands, to ensure a greater level of bio-compatibility the exposed metal placed on the outer capsule has been coated with a layer of alumina (Al_2_O_3_) (26). To deposit the Al_2_O_3_ layer, first a colloidal solution is prepared with the composition of 1.5 g Al_2_O_3_ powder, 20 ml 2-propanol, and 10 ml diethylamine. After that, the fabricated prototype is dipped into the prepared solution and the layer is grown over the antenna surface by a controlled dip coating technique (Apex Instruments, Xdip-XV1). Using this technique, the thickness of the alumina layer is controlled and a thin layer of coating is provided on the proposed capsule module. For further study, this conformal capsule module is placed inside a human torso model (Figure 6) in Ansys HFSS. Later, the fabricated prototype has been validated using a multilayer porcine slab. The simulated and measured return loss characteristics (Figure 7) of the capsule module are analyzed. In Figure 7, it is also shown that the inclusion of the alumina layer doesn't affect the return loss pattern of the antenna.

### Antenna SAR reduction

**Figure 8:**
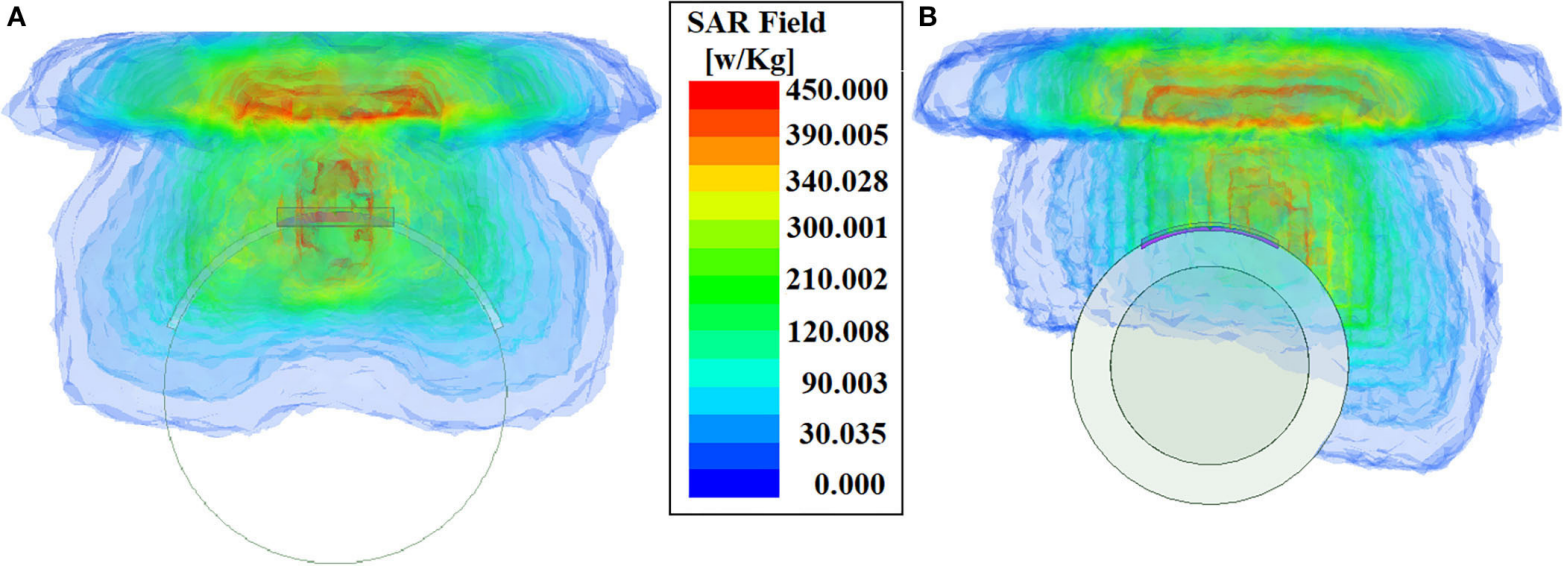
Average SAR distribution inside human muscle: **(A)** antenna without absorber metasurface and **(B)** antenna with absorber metasurface.

SAR is a measure of EM radiation absorbed by a unit mass of human tissue. Mathematically SAR of an implantable antenna over a unit volume of human tissue can be expressed as,


$$\begin{array}{l} SAR=\;\frac{{\sigma \;E}^{2}}{\rho } \end{array}$$

where σ is the conductivity of the human tissue where the antenna is placed, *E* is the electric field due to the antenna radiation and ρ is the density of the human tissue. So it can be seen that, for a fixed amount of EM radiation this absorption becomes the property of that tissue itself. Hence the existing techniques, those used for SAR reduction in wearable antennas, may cause a paradoxical situation in case of implantable antennas. Antennas, which are implanted inside the human body, radiate inside human tissue and make the tissue exposed to EM radiation. The use of EM shielding might reduce the SAR value by limiting the antenna radiation, it will certainly, reduce the antenna gain drastically. As, for implantable antennas, all the essential and spurious radiation necessarily goes through the human tissue. Hence, lowering SAR with the existing technique might reduce the antenna gain and efficiency extensively, so the antenna performance might be affected. To overcome this paradox, in previous literature (12), we have already reduced the SAR of an implantable antenna using a ferrite superstrate by reducing the discontinuities of the near *E* field. However, two major concerns were evident in that approach. Firstly, the use of a ferrite superstrate degrades the bio-compatibility of the implantable antenna system. Moreover, the use of a ferrite superstrate decreases the antenna gain significantly along with the SAR. The implantable antenna gain is usually found to be reduced due to the absorbing nature of human tissue, further decrease in antenna gain may restrict the efficient communication. Hence, in the present work, we are using for the first time, metasurface absorber to reduce SAR without affecting the antenna gain much. As the antenna under consideration is a CPW-fed antenna, significant amount of back lobe radiation can be experienced. However, this back lobe has not been used for communication and can be eliminated without degrading the desired antenna performance. Hence absorber metasurface, placed below the antenna, absorbs the back-lobe radiation to minimize the exposure of human tissue toward EM radiation. As we know, the SAR is a measure of power absorbed by the human tissue per unit of mass. By eliminating back radiation, we limit the amount of antenna radiation absorbed into human tissue. Thus, we control and reduce the average SAR value. The average SAR distribution of the antenna with and without metasurface absorber has been studied (Figure 8) to validate the proposed SAR-reduction technique. This figure shows that due to the presence of the metasurface absorber below the printed dipole antenna, the SAR distribution below the antenna has been immensely reduced. This is in parlance to the unique approach to reduce SAR by absorbing the undesired back radiation of the antenna, and thus the maximum amount of human tissue is not exposed to the EM radiation.

**Figure 9:**
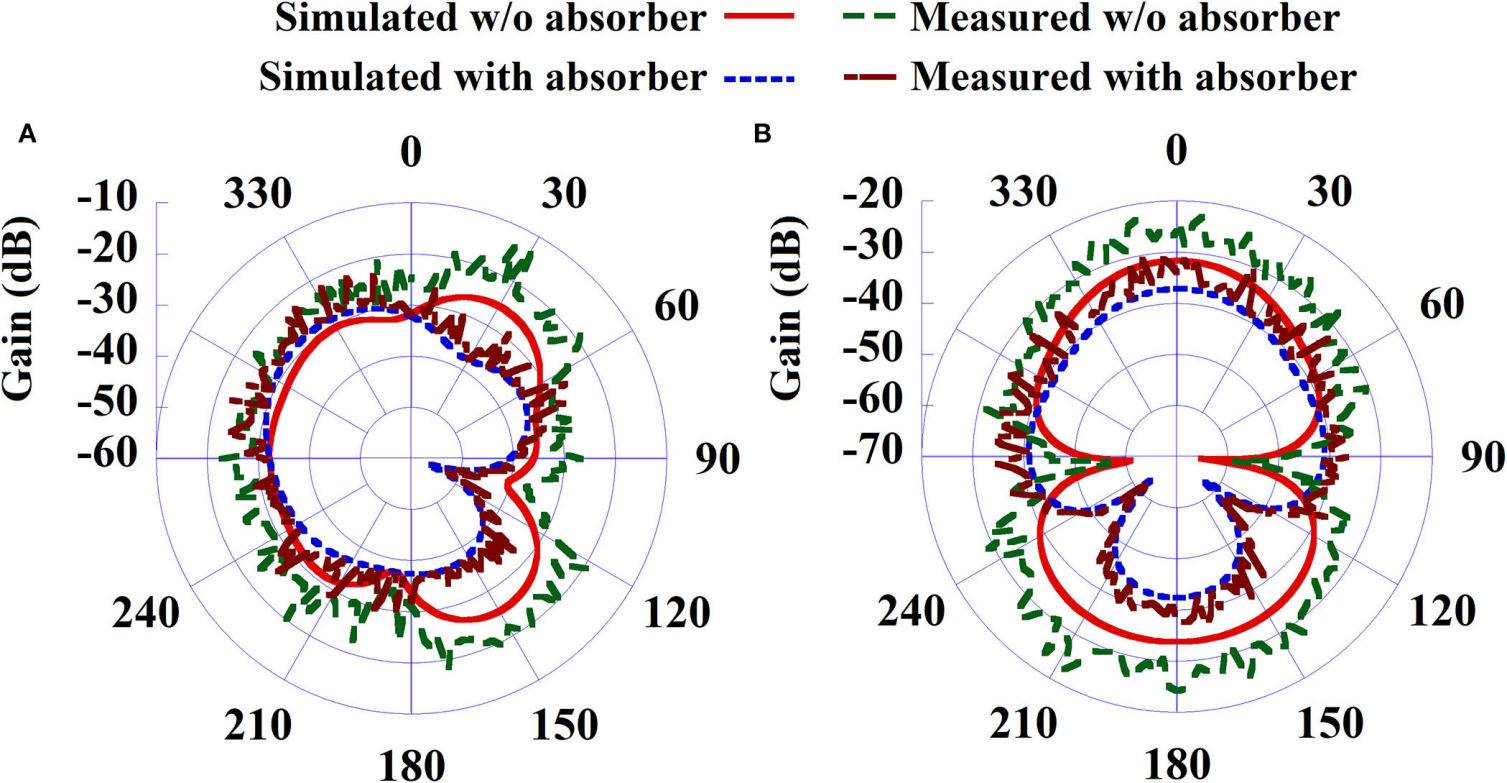
Simulated and Measured co-polarization radiation pattern comparison between the antenna system without and with the absorber metasurface; **(A)** XY plane and **(B)** YZ plane.

**Figure 10:**
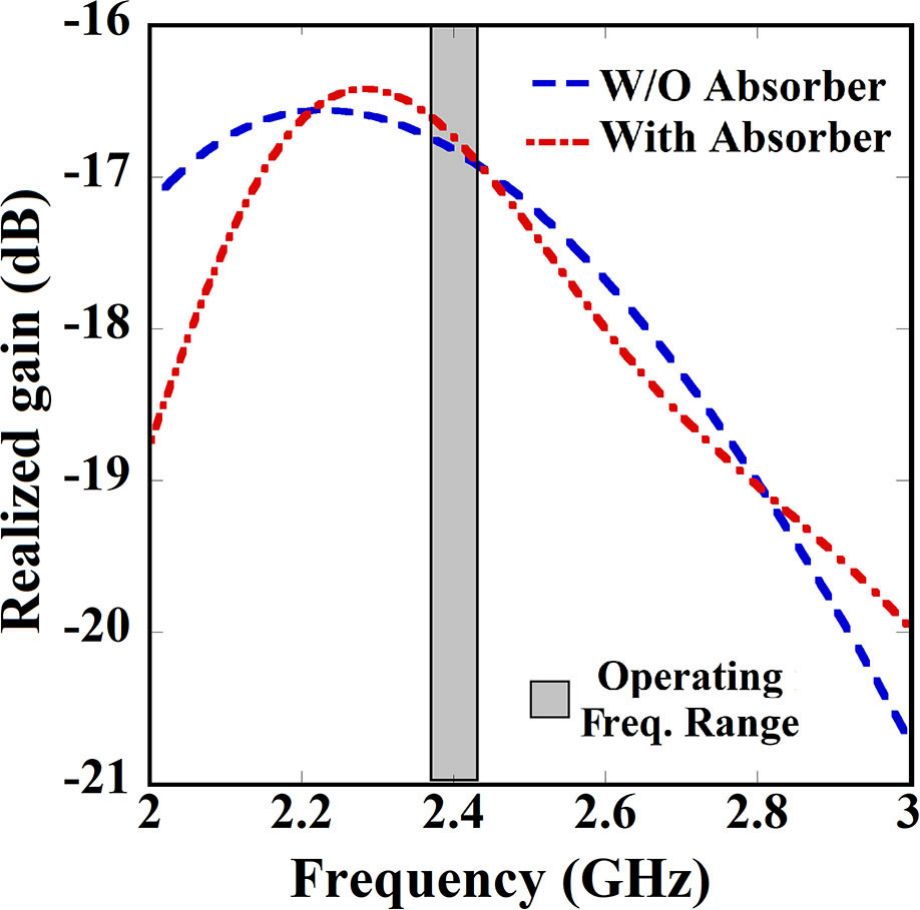
Peak realized gain over the frequency range for antenna module with and without absorber metasurface.

**Figure 11:**
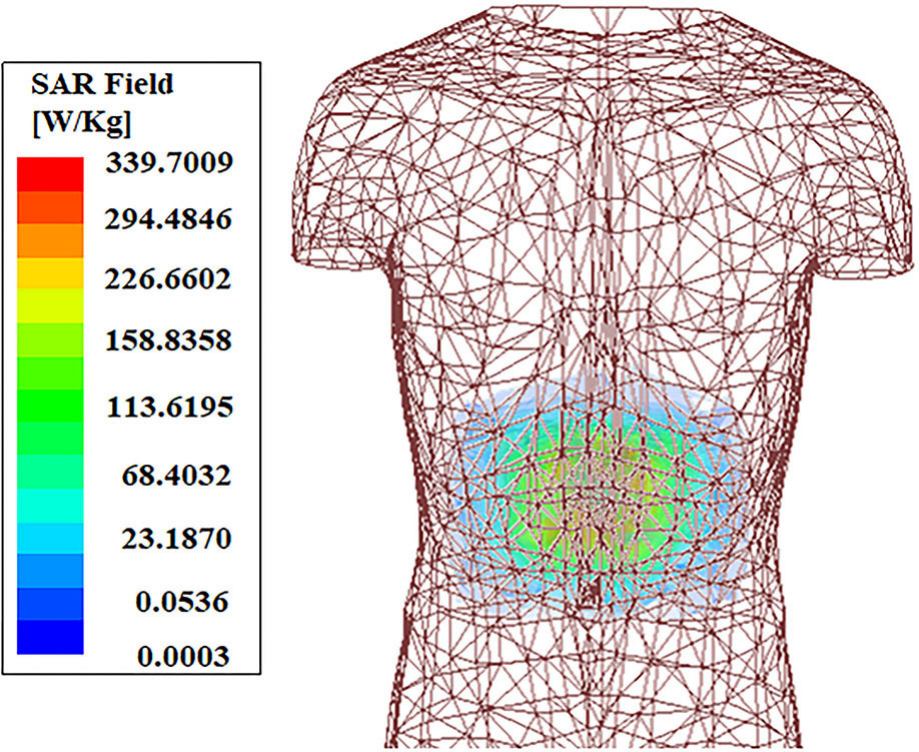
SAR distribution inside human torso.

Overall SAR value has been reduced by 24% approximately, as this technique almost eliminates the SAR due to unused back radiation without significantly affecting the antenna performance. Antenna gain (Figure 9) and efficiency remain almost unaffected by this SAR reduction technique. Figures 9A,B shows the simulated and measured co-polarized radiation pattern of the antenna-absorber capsule module. Figure 10 represents the peak realized gain over the frequency range of the proposed antenna module with and without the absorber metasurface. From the figure, it can be inferred that the inclusion of absorber metasurface does not affect the antenna gain. Furthermore, the 1 g average SAR distribution due to the capsule module inside the HFSS human torso model has been presented in Figure 11. Table 3 represents the comparative analysis of the measure of SAR reduction with different techniques in biomedical antenna applications. As SAR reduction technique for the implantable antennae is being proposed for the first time in this present literature, almost all the works that are being compared are for wearable antennas. Although in (12), we had successfully reduced the implantable antenna SAR by reducing the discontinuities of near E field using ferrite sheet, the antenna compromised its bio-compatibility. Hence, at present, the proposed technique with the capsule antenna-absorber module, is the unique approach for reducing the SAR value of an implantable antenna and with this technique, the peak SAR value has been reduced from 453 to 339.7 W/Kg (~24%) which is significant in the given condition. It may be seen that other non-implantable techniques for SAR reduction yields the better amount of reduction but those techniques cannot be used successfully in the case of implantable scenario. Hence, our proposed technique with absorber metasurface is the best possible solution to the problem regarding EM absorption due to in-body antennas. Also, this technique upholds the bio-compatibility as well as other antenna parameters intact.

**Table 3:** Comparative study of the SAR reduction techniques for biomedical antennas.

**References**	**Antenna**	**Freq. (GHz)**	**Technique**	**1 g SAR reduction**	**Remarks**
Augustine et al. (27)	Integrated inverted F antenna	2.4	Shielding using ferrite sheet	85.5%	Wearable application
Bhattacharjee et al. (28)	Meanderd antenna	2.4	E-field cancellation	25.5%	Wearable application
Das and Yoo (11)	Telephonic loop antenna	1.9	Reflection using EBG structure	24%	Wearable application
Wang et al. (29)	Micro-strip antenna	2.4	AMC structure	>70%	Wearable application
El-Atrash et al. (30)	Monopole antenna	3.5	Reflection using AMC array	99%	Wearable application
Mitra et al. (12)	Monopole antenna	2.4	Reducing the discontinuities of near E field using Ferrite sheet	42%	Fully implantable but ferrite sheet is not bio-compatible
This work	Capsule module antenna design	2.4	Absorption using metasurface	24%	Implantable and less-invasive unique technique

EBG, electronic band gap; AMC, artificial magnetic conductor.

## Result

**Figure 12:**
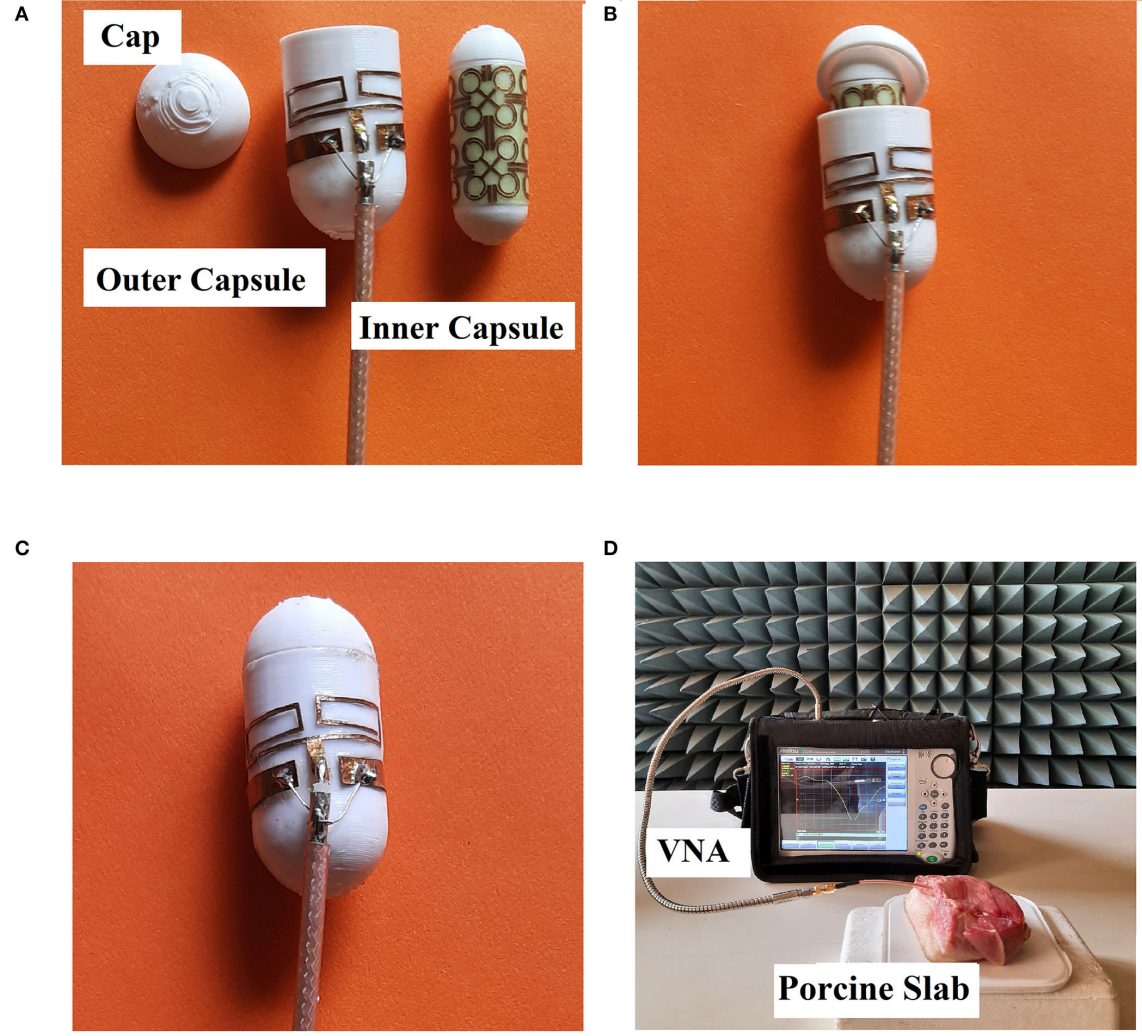
Fabricated prototype analysis: **(A)** capsule module parts, **(B)** arrangements of the module, **(C)** capsule module top view, and **(D)** *in vitro* test setup using porcine slab.

In the previous section, a printed dipole antenna was designed and analyzed. Also, an absorber metasurface has been proposed to reduce the SAR of that implantable antenna. Both the antenna and metasurface have been fabricated on a PLA substrate (Figures 12A–C). The proposed antenna-metasurface system is designed as an ingestible capsule module using PLA to make the entire system less-invasive. The inner capsule contains the metasurface printed around the outer surface. This is a closed capsule and the metallic structure has been pasted to create the absorber. This absorber is placed inside the outer capsule, which is open at one end. This outer capsule holds the antenna as well. The radiator and coplanar ground planes are cut from a copper sheet to be pasted around the outer capsule. The outer capsule is closed by using a PLA cap. This enclosed capsule having the antenna at its outer surface and inner capsule with absorber metasurface within, a porcine slab to test for various antenna parameters. This *in vitro* measurement has been carried out using a network analyzer (Anritsu S820E) and 50Ω SMA connector probes. We have used a porcine slab, as its dielectric properties are similar to those of the human tissue equivalent, to establish an *in vitro* setup using VNA (Figure 12D). These prototypes consisting of coaxial cable extensions are purely fabricated for *in vitro* analysis purposes. Measurement yields almost similar return loss characteristics compared to the simulated analysis (Figure 7). The efficiency and gain of the antenna remain unaffected even after the inclusion of the absorber metasurface (Figure 10).

## Discussion

In this paper, a unique technique has been proposed to reduce the SAR of the implantable antenna using absorber metasurface. The redundant back radiation of the printed dipole antenna has been absorbed by the metasurface to reduce the average SAR by 24%. The antenna-absorber system is designed as a compact capsule module to enhance the conformal nature of the system and also, make the system less-invasive. This capsule module can be attached to any implantable medical device as a transceiver unit. This ingestible capsule, made of PLA, is biocompatible in nature. The fabricated module has been tested *in vitro* using a multi-layered porcine slab. This technique helps us to reduce the SAR of the implantable antenna without affecting the antenna gain much. Also other parameters, such as antenna efficiency, impedance bandwidth, etc. remain almost unaffected due to the inclusion of absorber metasurface. Hence, this technique contributes to curtailing EM radiation absorption significantly, which results in biologically safer usage of implantable antennas. Using this technique the SAR of the proposed antenna has been reduced from 453 to 339.7 W/Kg. However, this reduced value is beyond the permissible value of the SAR according to FCC standards, i.e., 1.6 W/Kg. To attain that a well-used practice is to reduce the input power of the antenna. However, with the technique proposed here, we can reduce SAR value so that more input power can be given to the antenna for efficient communication. In this work, we can provide a maximum input power of 5.5 mW instead of 4.8 mW due to the incorporation of this proposed SAR technique. While this input power is sufficient to establish an in-body to an on-body communication link, a tradeoff between the higher input power and lower SAR value will always remain a key factor for implantable antenna design. In future development based on this literature, the maximum input power may be increased for better in-body communication. Table 4 shows the maximum input power allowed for some of the implantable antennas in the literature. Unlike our proposed work, these compared literatures are given the obtained SAR value and have not mentioned any SAR reduction technique.

**Table 4:** Comparative study of the SAR and maximum allowable input power for implantable antennas.

**References**	**Antenna**	**Freq. (GHz)**	**Maximum SAR (1W input power)**	**Maximum allowable input power**
Mahe et al. (14)	Dual ring slot	2.45 GHz	297 W/Kg	5.6 mW
Rajagopalan and Rahmat-Samii (15)	Meandered dipole	1.4 GHz	330 W/Kg	4.8 mW
Miah et al. (16)	Loop antenna	433 MHz	Not given	7.1 mW
Wang et al. (18)	Double layer patch	2.4 GHz	596.33 W/Kg	2.5 mW
Shah et al. (31)	Circular patch	1.4 GHz	368 W/Kg	5.43 mW
Shah et al. (32)	Meandered patch	915 MHz 2.45 GHz	377.6 W/Kg 279.5 W/Kg	3.7 mW 5.7 mW
This work	Capsule module printed dipole	2.4 GHz	339.7 W/Kg	5.5 mW

## Data availability statement

The raw data supporting the conclusions of this article will be made available by the authors, without undue reservation.

## Ethics statement

Ethical review and approval was not required for this study in accordance with the local legislation and institutional requirements.

## Author contributions

SD performed the design and experiments of the proposed antenna and wrote most of the article. AC designed and fabricated the capsule. DM and BM were involved in the critical analysis of the sensor's working. SD and BM were involved in the simulation software. RA has done the review, proofread, and provided antenna design techniques. RA and DM were involved in the supervision of the project, conception, design of the research, and approved the final version of the manuscript. All authors contributed to the article and approved the submitted version.

## Conflict of interest

The authors declare that the research was conducted in the absence of any commercial or financial relationships that could be construed as a potential conflict of interest.

## Publisher's note

All claims expressed in this article are solely those of the authors and do not necessarily represent those of their affiliated organizations, or those of the publisher, the editors and the reviewers. Any product that may be evaluated in this article, or claim that may be made by its manufacturer, is not guaranteed or endorsed by the publisher.
